# The Dutch Dystrophinopathy Database: A National Registry with Standardized Patient and Clinician Reported Real-World Data

**DOI:** 10.3233/JND-240061

**Published:** 2024-09-03

**Authors:** N.M. van de Velde, Y.D. Krom, J. Bongers, R.J.A. Hoek, N.A. Ikelaar, M. van der Holst, K.J. Naarding, J.C. van den Bergen, E. Vroom, A. Horemans, J.G.M. Hendriksen, I.J.M. de Groot, S.L.S. Houwen-van Opstal, J.J.G.M. Verschuuren, H.A. van Duyvenvoorde, R.R. Snijder, E.H. Niks

**Affiliations:** a Department of Neurology, Leiden University Medical Center, Leiden, The Netherlands; b Duchenne Center Netherlands, Leiden, The Netherlands; c Department of Orthopaedics, Rehabilitation and Physiotherapy, Leiden University Medical Center, Leiden, The Netherlands; d Duchenne Parent Project, Veenendaal, The Netherlands; e Spierziekten Nederland, Baarn, The Netherlands; f Kempenhaeghe Center for Neurological Learning Disabilities, Heeze, The Netherlands; g Department of Rehabilitation, Donders Center of Neuroscience, Radboud University Nijmegen Medical Center, Nijmegen, The Netherlands; h LUMC Biobank Organization, Leiden University Medical Center, Leiden, The Netherlands; i Department of Clinical Genetics, Leiden University Medical Center, Leiden, The Netherlands; j Department of Neurology, University Medical Center Utrecht, Utrecht, The Netherlands

**Keywords:** Duchenne muscular dystrophy, Becker muscular dystrophy, registry, real-world data, FAIR, trial readiness

## Abstract

**Background:**

Duchenne and Becker muscular dystrophy lack curative treatments. Registers can facilitate therapy development, serving as a platform to study epidemiology, assess clinical trial feasibility, identify eligible candidates, collect real-world data, perform post-market surveillance, and collaborate in (inter)national data-driven initiatives.

**Objective:**

In addressing these facets, it’s crucial to gather high-quality, interchangeable, and reusable data from a representative population. We introduce the Dutch Dystrophinopathy Database (DDD), a national registry for patients with DMD or BMD, and females with pathogenic *DMD* variants, outlining its design, governance, and use.

**Methods:**

The design of DDD is based on a system-independent information model that ensures interoperable and reusable data adhering to international standards. To maximize enrollment, patients can provide consent online and participation is allowed on different levels with contact details and clinical diagnosis as minimal requirement. Participants can opt-in for yearly online questionnaires on disease milestones and medication and to have clinical data stored from visits to one of the national reference centers. Governance involves a general board, advisory board and database management.

**Results:**

On November 1, 2023, 742 participants were enrolled. Self-reported data were provided by 291 Duchenne, 122 Becker and 38 female participants. 96% of the participants visiting reference centers consented to store clinical data. Eligible patients were informed about clinical studies through DDD, and multiple data requests have been approved to use coded clinical data for quality control, epidemiology and natural history studies.

**Conclusion:**

The Dutch Dystrophinopathy Database captures long-term patient and high-quality standardized clinician reported healthcare data, supporting trial readiness, post-marketing surveillance, and effective data use using a multicenter design that is scalable to other neuromuscular disorders.

## INTRODUCTION

Duchenne and Becker muscular dystrophy (DMD and BMD) are rare progressive X-linked neuromuscular disorders with an estimated incidence of around 1 in 5.000 and 1 in 20.000 live male births respectively [1–3]. Both dystrophinopathies are caused by pathogenic variants in the *DMD* gene. These result either in lack of the dystrophin protein (DMD), or normal/ reduced expression of a partially functional dystrophin protein (BMD) [4, 5]. DMD is the most severe form of dystrophinopathy, with muscle symptoms manifesting in early childhood, ultimately resulting in fatal cardiac and pulmonary complications [6, 7]. BMD is a milder form of dystrophinopathy, in which the age of onset of symptoms and severity vary considerably [8, 9]. However, no diagnostic criteria can fully distinguish between both forms, and dystrophinopathies should rather be considered a spectrum. While the disorders primarily affect males, females with a heterozygous pathogenic *DMD* variant can also have clinical manifestations [10, 11].

Currently no curative treatment is available, but life expectancy in DMD has been significantly improved by multidisciplinary care, respiratory support, chronic use of corticosteroids, and cardioprotective treatment [7, 12]. Furthermore, numerous therapeutic strategies have emerged for DMD [13], and recently, also for BMD [14]. The design and execution of these randomized controlled studies has been complicated by the rarity of either disease. Additionally, the high phenotypic variability and the complicated trajectory to develop sensitive and relevant outcome measures have added further challenges. Detailed understanding of the natural history has proven to be crucial [15], but prospective research protocols suffer from poor recruitment and the risk of selection bias [10]. Because of the limited duration of most clinical study protocols [10], it is difficult to establish correlations between longitudinal functional parameters and important disease milestones such as loss of ambulation [16–18].

Continuous collection of real-world data (RWD) is an alternative approach to study the natural history. Recently, it has been illustrated that the utilization of RWD in assessing outcome measures such as the North Star Ambulatory Assessment (NSAA) and Six-minute Walking Test (6MWT) can serve as a valuable approach for baseline adjustment of prognostic factors and for evaluating the efficacy of drugs in ambulatory individuals with DMD [19, 20].

RWD require standardized high-quality assessments, and can be facilitated via an advanced data management system. The use of a national patient registry as a platform to collect RWD has several advantages. It provides epidemiological data, and allows collaboration in (inter)national data-driven initiatives. With sufficient coverage, it also facilitates comparison of pertinent characteristics of participants and non-participants in clinical studies, and thus assess whether the studied cohort is representative of the national population [21].

The initial iteration of the national registry for patients with DMD, BMD or females with a heterozygous pathogenic *DMD* variant in the Netherlands, named Dutch Dystrophinopathy Database (DDD), was established in 2008 as a stand-alone database at the Leiden University Medical Center (LUMC) [22]. In 2014, second iteration of DDD was upgraded to a web-based database management system developed at the LUMC [23]. In 2016, the Duchenne Center Netherlands (DCN) was founded as collaboration between three academic partners (LUMC, Radboudumc and Kempenhaeghe-MaastrichtUMC+) and two patient organizations Duchenne Parent Project and Spierziekten Nederland, with financial support from Spieren voor Spieren foundation. Objectives of DCN were to increase trial readiness, to facilitate multicenter collection of standardized longitudinal RWD, and to improve governance and quality control. These goals prompted a re-evaluation of the existing database, resulting in an overhaul of its structure, as well as its corresponding governance, including personnel.

The new implementation comprised curated and semantically annotated data elements aligned with international care guidelines. A new hosting platform was chosen to ensure ongoing compliance to ISO 27001. To ensure quality and quantity of data, overall operations and continuity, a project coordinator and a database manager were appointed. These modifications culminated in the establishment of the current, third iteration of, now multicenter, DDD in 2018.

The current registry has a dual nature: encompassing both self-reported information via questionnaires for patients nationwide, and clinician reported healthcare data from patients visiting the DCN’s academic expert centers. The Dutch healthcare system includes seven University Medical Centers that have specialized clinics to provide care for children and adults with any neuromuscular disease, but each center focuses on specific subtypes as the primary area of their research. Most dystrophinopathy patients are therefore seen within the centers of DCN, but standards of care are shared amongst all centers.

This report describes the new structure, governance, and population of the current third version of DDD, serving as an example of how healthcare data can be effectively (re)used when captured in a standardized manner.

## MATERIAL AND METHODS

### Consent and ethical approval

All Dutch patients diagnosed with a dystrophinopathy, and females with a heterozygous pathogenic *DMD* variant are eligible to be registered in the DDD. Registration can be completed by filling out an age-appropriate informed consent online or on paper. To maximise participation to the registry, patients can give their consent to five different levels of inclusion (Table 1). The minimal requirement for registration is level one, consent to provide name, date of birth, contact details, and the clinical diagnosis, and allowing to request genetic test results from the treating physician. The remaining four registration levels are non-compulsory. Level two includes filling out a yearly questionnaire about disease milestones and medication use. Level three asks consent to store the clinical data that was collected as part of regular care at one of the academic centers of DCN. Level four and five comprise exchange of coded (non-aggregated) data with non-commercial and commercial partners, respectively. The medical ethical committee of Leiden-Den Haag-Delft declared that the registry is not subject to the Medical Research Involving Human Subjects Act (WMO). The medical ethical committees of Radboudumc and Kempenhaeghe-MUMC have declared the local feasibility of this registry. The process of setting up the documents for DDD iteration 3, including the protocol, patient information forms, informed consent, and the declaration from the ethics committee, took approximately 8 months. The declaration was received in August 2019.

**Table 1:** DDD registration levels

Level	Consent	Objectives
1.	Contact details Clinical diagnosis Request of genetic test results	Approach of potential participants for clinical studies without pre-screening Ongoing study of epidemiology Provide feedback on use of the registry.
2.	Yearly questionnaire on patient-reported disease milestones and use of medication	Epidemiology Approach of potential participants *with* pre-screeningStudy of natural history using patient reported data
3.	Storage of clinical healthcare informationfor patients visiting one of the clinical centers of Duchenne Center Netherlands	Study of natural history using patient and clinician Re-use of clinical data for investigator-initiated studies
4.	Exchange coded data with non-commercial partners	(Inter)national collaborations with academic partners
5.	Exchange coded data with commercial partners	(Inter)national collaboration with commercial partners

### Registry governance

The procedure for registration of patients and data collection was compliant with the most recent version of EU General Data Protection Regulation (GDPR) [24], which, crucially, entails separate storage of personal (identifiable) and clinical data.

In November 2018, DCN set up a general board for the third version of DDD that consisted of representatives of each of the five aforementioned partners: LUMC, Radboudumc, Kempenhaeghe-MUMC+, and the two patient organizations, i.e., Duchenne Parent Project and Spierziekten Nederland. Furthermore, a collaboration agreement was drawn up between these five partners that described the allocation of tasks and responsibilities related to the design, management and use of the database. For example, LUMC, Radboudumc and Kempenhaeghe are jointly responsible for the GDPR-compliant storage and use of personal data. In addition, all parties have entrusted the design and management of DDD to LUMC and discuss, at least annually, the status of the database. These discussions cover both design and use of the data, the collaborations with third parties, and (research) projects involving DDD data. Part of the collaboration agreement was setting up a DDD advisory board with independent representatives from each of the five DCN partners. The advisory board is a consultative body, making solicited and unsolicited recommendations on scientifically relevant aspects regarding the purpose and use of DDD, and evaluates data requests from researchers to provide advice to the DDD general board.

### Technical design of the registry

To favor interoperability and standardization of the collected healthcare data, in 2018 the registry was re-designed using an information model based on the FISMA-model (Framework for Information Specification Modelling and Architecture). FISMA is a direct descendant of the information model used by the Dutch national biobanking initiative Parelsnoer (aptly named PRISMA) set up by the Dutch University Medical Centers [25]. FISMA is a system-independent information model that adheres to clinical guidelines and is based on detailed clinical models (ISO/TS 13972:2015). These models are evidence-based, comprising data elements that are annotated with meta-information (like ICD-10/ICD-11, ATC, HPO, SNOMED Clinical Terms). In this respect, FISMA is considerably more extensive than its predecessor PRISMA – and as a whole, adheres to FAIR-principles [26]. As part of its comprehensive framework, the DMD-specific FISMA contains metainformation about, references to, and conversions for TREAT-NMD data elements [27]. As FISMA is inherently system-independent, it governs the design process of data capture solutions, resulting in successful implementations in HiX, the electronic healthcare system (EHS) at LUMC and in Castor EDC, a secure web-based data management system in 2020 [28]. The data collected from either system is thus interoperable, exchangeable, and directly suitable for analysis purposes. Upon request, data extracted from EHS can even be merged with data extracted from CastorEDC without recoding and only minor structural transformations. An implementation in EPIC, the EHS at Radboudumc, is currently underway.

Castor EDC (ISO 27001, NEN7510, ISO 9001) is used as the web-based data platform for the current version of DDD. This data platform is suitable for multi-center studies, allows direct entry of data, provides multilevel access for operators and data encryption at multiple levels. Furthermore, it includes an audit trail, data quality control measures such as auto-calculations, missing value flags and appropriate ranges. Most importantly, its data-entry interface requires minimal to no training. Especially for the functional outcome measures, several checks, restraints, and calculations for sub-scores, as well as automated entry of earlier outcomes were realized to increase accuracy. Two-factor authentication is enforced to access this system.

### Affirmation of consent

In the first version of DDD, 462 patients or their caregivers, provided consent between 2008 and 2014, via the method as previously published [22]. For the second version, an additional 183 patients or their caregivers, gave their consent for registration between 2014 and February 2020. In the first quarter of 2020, each participant of the previous versions of DDD was asked by letter whether they would like to be registered in third and current version of DDD. They received a new informed consent form (ICF), registration forms, and an information leaflet summarizing the additional purposes and adjustments. Non-responders were followed-up by telephone. Those who were successfully contacted and gave their approval by telephone, received a new set of forms. After four and eight months, another attempt was made to reach those who still had not responded. Hereafter, non-responders were included in the third version of DDD as part of the no-objection system of the Dutch Medical Treatment Contract Act (WGBO), under the assumption that consent could not be obtained due to incorrect contact details, or due to the possibility that the participant had deceased. The non-responders and deceased cohort adhered to their original consent preferences. This included agreeing to store their contact details and medical healthcare data, completing a questionnaire, and optionally allowing the anonymized use of their data for TREAT-NMD. Modality (written, oral or transfer of non-responders) and version of the relevant informed consent were registered per participant.

### Enrollment of new patients

To reach out to unregistered patients and increase coverage, newsletters about the registry were sent out in July and October 2020 by the two associated patient organizations. In addition, ready-for-use ICFs were published on the DCN website [29], and sent out to clinicians who treated patients diagnosed with dystrophinopathy, and were employed at the seven Dutch UMCs, of which the four associated CHMVs are part of [30]. Information about the new version of DDD was also shared during national meetings for caregivers of patients with neuromuscular diseases, and national patient days. Lastly, an information letter was added to all diagnostic reports from Leiden Diagnostic Genome Analysis Laboratory (LDGA). This laboratory has been the *de facto* Dutch expertise center for the genetic screening of *DMD*, including validation and/or confirmation of a pathogenic *DMD* variant detected in any other laboratory in the Netherlands. Any information pertaining to diagnosis or genetics supplied by all patients in DDD or their healthcare providers, was validated by the clinical laboratory geneticist of aforementioned LDGA. For patients visiting one of the DCN centers, the clinical diagnosis was evaluated by clinicalexperts.

### Data collection

All clinician-reported data that had been collected in the previous two versions of DDD were converted to match the defined data elements in the third version of DDD between November 2020 and June 2021. Among the converted data was information regarding milestones, corticosteroid use, medication, data from physical examinations, cardiac and lung function assessments.

In the current version of DDD, both patient-reported and clinician-reported data are collected. If patients consent to level two, they receive yearly digital questionnaires. The questionnaires were designed with tailored questioning for BMD, DMD, and females with a pathogenic *DMD* variant, and capture aspects related to disease progression and feasibility-assessment for clinical studies. Subjects covered include diagnostic procedures, medication use, and disease milestones (e.g., loss of ambulation, the ability to raise a full glass to the mouth using one hand, and the start of respiratory support).

An overview of the questionnaires for DMD and BMD are provided online at ‘FISMA, the information framework & model’ [31]. The questionnaires are sent out yearly via email using CastorEDC’s scheduling features, followed by three reminders sent with a two-week interval. To minimize participants’ effort, the questionnaires that follow-up the first questionnaire, only queries information on items that may change over time (e.g., medicine use and disease milestones).

The clinician-reported data (level 3) are collected as part of regular care for participants who have their outpatient visits at either academic center, Radboudumc or LUMC. The collected data is based on the international guidelines for DMD published in 2018, and in agreement with the extended Duchenne Core Data set published by TREAT-NMD on their website in 2021 [27, 32–34]. The main directive for this longitudinal data set is that it has to be sustainable, relevant and standardized.

The variables in the database were therefore structured in three different ways; 1) one-time entries, 2) multiple measurements during outpatient visits, or 3) continuously, without an explicit association to a clinical observation. Achieving certain milestones is an example of a one-time entry in a progressive disease. Physical examination and functional assessments are examples of parameters that are collected during outpatient visits. Corticosteroid use (type, dosage, schedule) is captured continuously, thus allowing to estimate the total accumulated dose of corticosteroid use over any period of time.

To improve quality and ensure standardized data collection of the functional outcome measures, physiotherapists and occupational therapists within the affiliated centers all received uniform training. In addition to data collected during outpatient clinical visits, DDD also registers information about clinical trial participation, including start and stop date and, if relevant, leg of the trial, i.e., whether patients have been on placebo or active treatment. An overview of the type of data collection as well as the structure of data storage is shown online at ‘FISMA, the information framework & model’ [31].

### Data access

To gain access to data, any researcher, whether they are employed by one of the partners of DCN, or employed elsewhere, has to provide a written application. This application include the project leader’s name, the primary researcher, study rationale, research question(s), the potential impact for healthcare professionals and patients, as well as the study’s design and methodology. This application is reviewed by the advisory board first, and then by the general board. Upon unanimous approval, the applicant receives the requested coded data from the database manager. The DCN coordinator monitors and coordinates all involved communication, archiving and summarizing the responses, advice and communicating the decisions.

To contact and recruit eligible patients via DDD for an investigator-initiated study, the application is also seen and approved by the advisory- and general board. Upon approval, either the database manager or the DCN coordinator informs eligible patients via email. When interested, patients will be referred to the involved researcher. Researchers who are employed by the participants of DCN, after approval, will get access to the contact details of the eligible participants during the recruitment phase of the study.

In the Netherlands, all pharmaceutical clinical trials for DMD and BMD are conducted by the DCN centers. When such a trial has been approved by the institutional review board, the database manager provides personal contact information of eligible patients to the trial coordinators for recruitment for the duration of the inclusion process.

## RESULTS

**Fig. 1:**
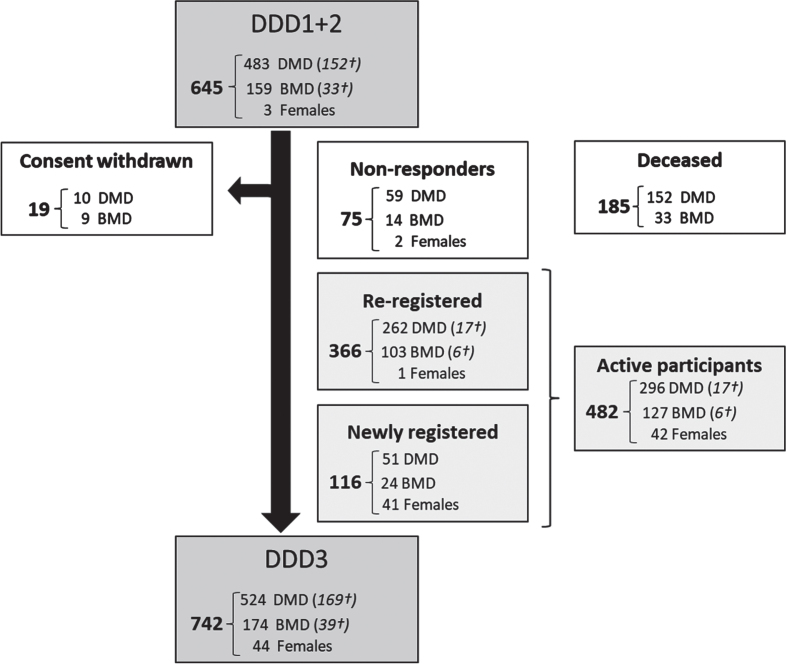
Flowchart of the number of Dutch participants with Duchenne, Becker and females with DMD mutation (re-)registered in the third version of DDD. Data analyzed per November 1, 2023. DDD: Dutch Dystrophinopathy Database, DDD1: first version in 2008. DDD2: second version in 2014. DDD3: third and current version since 2020. DMD: Duchenne muscular dystrophy, BMD: Becker muscular dystrophy. Females: Females with a pathogenic DMD variant. †Number registered as deceased.

In February 2020 a total of 645 patients were registered in the second version of DDD. In the third version, 366 of the participants from previous versions re-registered, 116 were newly registered participants, 75 were non-responders and thus implied consent from previous registration and 208 had passed away. Nineteen participants previously included explicitly declined consent for the new DDD (Fig. 1).

**Fig. 2:**
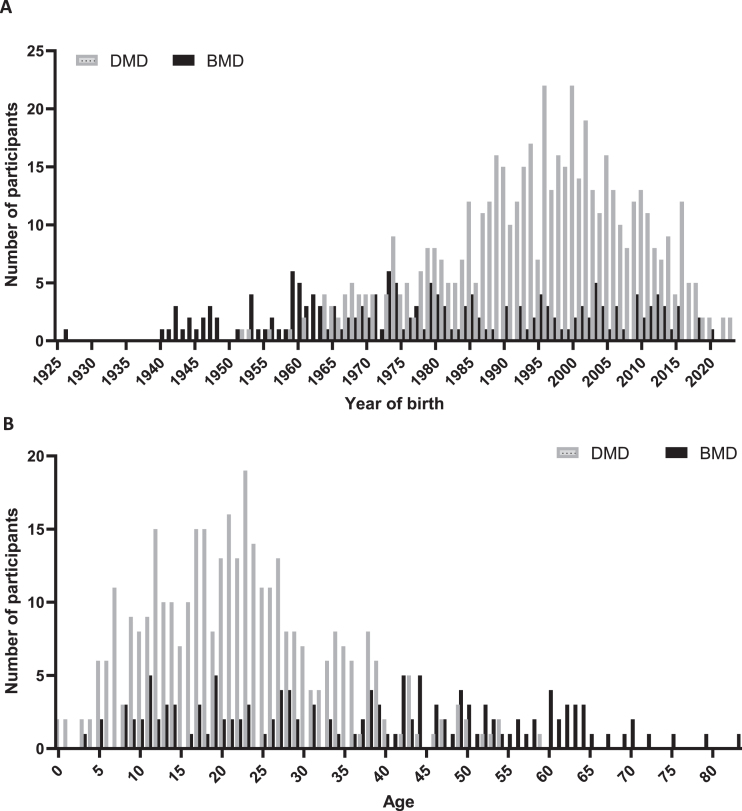
Demographic representation of registrants in the third version of DDD. In panel A, data are categorized by year of birth for 524 DMD (grey bars) and 174 BMD registrants (black bars). In panel B current age is shown for 355 DMD and 135 BMD participants registered as alive on November 1, 2023.

After re-evaluation the consent of existing participants and inclusion of new patients, 742 patients were included in DDD on 1 November 2023, comprising 524 participants with DMD, 174 participants with BMD and 44 females with a pathogenic *DMD* variant. Of these 742 patients, 169 clinically diagnosed with DMD and 39 diagnosed with BMD had passed away. Information on the age at death was available for 72 individuals with DMD and 21 with BMD, of whom genetic variant information was recorded for 66 with DMD and 18 with BMD. The average age at death for these 66 DMD participants was 25.8 years (SD 6.8), whereas for the 18 BMD participants, it was 49.5 years (SD 17.3). To present a comprehensive insight of coverage across various years, we have created a frequency diagram of the count of inclusions per year of birth for all 524 DMD and 174 BMD registrants and graphed the age for 355 DMD and 135 BMD participants registered as alive, as depicted in Fig. 2A and 2B. The counts of inclusion per year of birth span from 0 to 23 for DMD and 0 to 6 for BMD participants. The median age of 355 DMD, 135 BMD participants and 44 females with a pathogenic *DMD* variant was 21, 38 and 47 years respectively.

A genetic report was available for 659 out of 742 participants (88.8%, Table 2). Among patients with DMD, the majority, 304 (63%) had deletions in the DMD gene, of which 286 (94%) were out-of-frame deletions. Of the remaining DMD patients, 95 (12.2%) had duplications, 53 (11.0%) presented nonsense mutations, 37 (7.7%) showed frameshift mutations and 28 (5.8%) had splice-site variants within the DMD gene. In patients with BMD, the majority, comprising 108 (71.5%) patients, also had deletions in the DMD gene, with 100 (92.6%) of these being in-frame deletions. Of the remaining BMD patients 22 (14.6%) had duplications, 9 (6.0%) presented splice-site variants, 5 (2.6%) showed frameshift mutations and 3 (2.0%) had nonsense mutations within the DMD gene. In females carrrying a pathogenic DMD variant, deletions were also most prevalent, observed in 17 (68%) individuals, with a relatively equal distribution of in-frame versus out-of-frame deletions, namely 8 (47.1%) versus 9 (52.9%). For 83 participants, 41 DMD, 23 BMD and 19 females with a pathogenic DMD variant, no genetic report was available.

**Table 2:** Genetic data of participants in the third version of DDD

		Diagnosis			Total	
		DMD	BMD	Females	Subtotal	Total
Genetic report available -*n* (%)		**483 (92.2)**	**151 (86.8)**	**25 (56.8)**		**659 (88.8)**
	Deletion (%)	**304 (63.0)**	**108 (71.5)**	**17 (68.0)**		**429 (88.8)**
	In-frame*	18	100	8	120
	Out-of-frame*	286	8	9	294
	Duplication (%)	**59 (12.2)**	**22 (14.6)**	**2 (8.0)**		**83 (12.6)**
	In-frame*	10	21	–	30
	Out-of-frame*	49	1	2	50
	Small variants (%)	**118 (24.4)**	**18 (11.9)**	**6 (24.0)**		**142 (21.5)**
Variant type	*Frameshift*	37	4	4	35
	*Nonsense*	53	3	2	58
	*Missense*	0	1	–	6
	*Splice-site*	28	9	–	34
	*Other*	1
	Unidentified variant (%)	**2 (0.4)**	**–**	**–**		**2**
	No pathogenic variant detected (%)	**–**	**3 (2.0)**	**–**		**3**

Data analyzed per November 1, 2023. DMD: Duchenne muscular dystrophy, BMD: Becker muscular dystrophy, Females: Females with a pathogenic *DMD* variant. SD: Standard Deviation. *Predicted influence on the reading frame.

### Registration levels

**Fig. 3:**
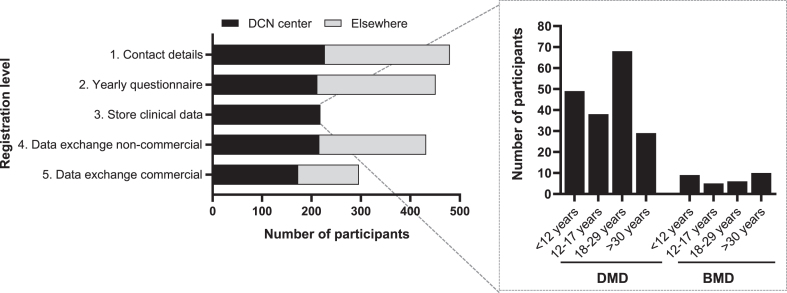
Number of participants per registration level within the third version of DDD. Black bars represent the participants who have their outpatient visit at one of the DCN centers (Radboudumc or LUMC). Gray bars represent the participants who don’t visit a DCN center. The inserted graph shows the number of participants with DMD or BMD who consented storage of their healthcare data, categorized per age. DMD; Duchenne muscular dystrophy, BMD; Becker muscular dystrophy, DCN; Duchenne Center Netherlands.

The 482 patients who actively consented for the third version of DDD participated at different levels in the registry (Fig. 3). A total of 451 (93.6%) participants provided consent for the yearly questionnaire (registration level 2). These included 291 participants with DMD, 122 with BMD, and 38 females with a pathogenic *DMD* variant. Of the 482 actively registered patients, 227 (47.1%) had their regular outpatient visits at Radboudumc or LUMC. Among these, 218 (96.0%) gave consent to store clinical data collected as part of regular healthcare (registration level 3) comprising 182 (83.5%) participants with DMD, 30 (13.8%) with BMD, and 6 (2,7%) females with a pathogenic *DMD* variant. Of the 482 DDD participants, a total of 432 (89.6%) consented to registration level 4, involving data exchange with non-commercial parties. In contrast, 296 (61.4%) consented to registration level 5, which entails data exchange with commercial parties (Fig. 3).

### Clinical characteristics

Clinical characteristics of 218 participants who consented to storage of their health data (consent level three) are presented in Table 3. As of November 1, 2023, 129 (72.8%) participants with DMD were taking corticosteroids (87 prednisone, 38 deflazacort, 4 vamorolone). Most of the prednisone and deflazacort users were on an intermittent steroid schedule (*n* = 120, 96%). Conversely, only one patient with BMD (3.3%) used an intermittent regime of steroids. On 1 November 2023, 132 (75,5%) of DMD and 3 (10%) of BMD participants were non-ambulant, with ‘loss of ambulation’ being defined as ‘walking less than 5 meters indoors without aids and support’. Average age of loss of ambulation was 11.1 years (SD 2.6) for DMD and 37.0 years (SD 19.9) for BMD participants.

**Table 3:** Characteristics of 218 participants consenting to use their healthcare data

	Diagnosis		
	DMD	BMD	Females
*Age categories (n)*
<12	47	9	–
12–18	38	5	–
18–30	67	6	1
>30	25	10	5
Alive/Deceased	177/5	30/	6/0
*Ambulation status*
Ambulant (*n*)	50	27	6
Non-ambulant (*n*)	132	3	–
Age at loss of ambulation	11.1±2.6	37.0±19.9	–
*Mean age*±*SD in years*
*Corticosteroid users (n)*
Total steroid-users	129	1	–
Prednisone	87	–	–
*Daily*	*2*
*Intermittent*	*84*
*Unknown*	*1*
Deflazacort	38	1	–
*Daily*	*2*	–
*Intermittent*	*36*	*1*
Vamorolone	4	–	–
Missing	1
No steroid-users	48	29	5
Total	177	30	6

Loss of ambulation defined as unable to walk more than 5 meters indoors without aids and support. DMD; Duchenne muscular dystrophy, BMD; Becker muscular dystrophy, Females; Females with a pathogenic variant in the *DMD* gene.

### Inform patients and access of data for clinical studies

Between the renewal of DDD in February 2020 and November 2023, eligible patients were informed about nine pharmaceutical trials and five investigator-initiated studies that were conducted by researchers employed by participating centers of DCN. DDD was also used to collect, and report standardized clinical assessments for the conditional reimbursement of ataluren in the Netherlands in collaboration with the National Health Care Institute. In addition, seven TREAT-NMD enquiries have been carried out to check feasibility. Twelve data requests by researchers employed by partners of DCN have been approved to use coded data for clinical studies[35–37].

From 2023 onwards, participants receive semi-annual digital newsletters with information about the included cohort of DDD, information on its use, results of the observational studies conducted with data from DDD, and an overview of public results of the pharmaceutical clinical studies performed within DCN. These are published on the Dutch Duchenne Center website as well [38, 39].

## DISCUSSION

In this manuscript, we describe the development, structure, governance, and content of the Dutch Dystrophinopathy Database (DDD), a national registry for patients with a dystrophin-related muscular dystrophy, and females with a heterozygous pathogenic *DMD* variant. The underlying framework of the third version of DDD ensures generation of interoperable, exchangeable, standardized, longitudinal data, captured within its clinical context, by enabling the (re)use of Real-World healthcare data. With this, DDD serves as a platform to study the epidemiology and natural history in the Netherlands. It facilitates conducting investigator-initiated studies, pharmaceutical trials, and post-market surveillance. This third version was designed to introduce different levels of participation, to include patient and clinician reported data, to comply with regulatory requirements on data handling, to optimize transparency in use of data, and to provide regular feedback to participants.

For any registry of rare diseases, it is crucial to ensure national coverage. We tried to maximize inclusion by multiple queries for eligible patients in all academic centers in the Netherlands since 2008, by repeatedly asking patients to enrol via patient organizations, by adding information on the registry in genetic reports, and most recently, by providing the possibility for online consent.

By offering the possibility to participate on several levels with only diagnosis and contact details as minimal requirement, we have attempted to remove any obstacle to participate in the registry. Based on current epidemiological estimates of the global prevalence of DMD, around 2.8 per 100,000 inhabitants [40], one would predict approximately 500 DMD patients to be alive in the Netherlands, given its current population of 17.8 million inhabitants. Given that number, DDD would currently cover around 70% of all DMD patients in the Netherlands. However, these numbers should be interpreted with caution, due to the worldwide variability in epidemiological data and survival, as well as potential changes in incidence over time due to increased awareness and counselling, and improved, more readily available diagnostics. An ongoing epidemiological study in the Netherlands aims to combine data from various sources such as hospital coding systems and data from all clinical genetic laboratories, which could help to provide an improved estimate of the coverage rate of the registry [41]. It should be noted that both the distribution of pathogenic variant types, as well as the age-distribution of included males, in our registry largely resemble the descriptive of previously described international cohorts [42–47].

The inclusion of females in DDD is notably low, considering that approximately two-thirds of all mothers of affected males are heterozygous carriers of the pathogenic variant [48]. This is primarily due to lack of active recruitment, as well as awareness of the possibility of and relevance for females to register in DDD, which has recently been emphasised in an international workshop [49]. Although at present we were able to validate only 56.8% of the genetic reports, we presume that these will be available for all registered females in the near future. We also aim to increase the inclusion of these women through proactive recruitment and education and gain more insights into the prevalence, clinical manifestations and psychosocial considerations through yearly questionnaires.

Disease registries are also an essential point of access to RWD which become increasingly more important in drug development, for example when used as external controls in clinical trials or to assess long-term efficacy and safety. A large-scale multi-institutional comparison on DMD between placebo data of multiple trials, and RWD of several observational studies, demonstrated that the motor assessments in both sets were highly comparable [19, 20, 50]. The ultimate goal would be to obtain high-quality clinical meaningful interoperable RWD with minimal burden for patients and clinicians. In DDD, the collected clinical data elements were based on the international standards of care, involved extensive discussion with healthcare practitioners, were aligned with the expanded data set of TREAT-NMD, and include common data elements for rare diseases registration from EU projects, EUCERD Joint Action, EPIRARE and RD-Connect [27, 32–34, 51].

DDD, along with its contextual metadata, contributes to attain high-quality valuable RWD in several ways. First, the functional assessments in the DCN centers are performed by experienced physiotherapists in the same protocolized fashion as when conducting clinical trials. This minimizes center-to-center and intra-examiner variations, and thus results in increased consistency, accuracy and quality of the data. Second, the genetic records undergo validation by the clinical laboratory geneticists at LDGA. Third, the data elements in DDD can easily be adapted and extended as new insights evolve, or as care guidelines change. Fourth, the current implementation of standardized and uniform registration in EHS facilitates registration at the source, thereby reducing the effort needed to capture RWD and minimizing data entry errors.

Registration level 3 of DDD, clinical data collected as part of regular healthcare, also enables post marketing surveillance, as shown by the example of ataluren. The registry is important for future reimbursement processes, especially for high cost, single-administration therapies such as gene transfer. An ongoing gap analysis aims to provide an overview of the consistency of the data obtained in the clinics and registered in DDD for patients who give their consent to use their health data.

DDD also facilitates studying the natural history of the national cohort, by including patients outside DCN, via the questionnaires in level 2. This is important when assessing inclusion bias in clinical studies [21]. It provides potentially more detailed information on clinical milestones. For example, in most RWD studies, loss of ambulation is assessed during a clinical visit and the accuracy of these data thus highly depend on the frequency and consistency of outpatient care. In most natural history studies, and even placebo arms from clinical trials, it has been difficult to obtain detailed data on medication use, and particularly on corticosteroid dosing and regimes. Both the online questionnaire, as well as extraction of pertinent information from the EHS, allow estimating the cumulative exposure to corticosteroids, which is known to have a large effect on the clinical course of the disease.

Trial readiness is critical to support new therapy developments for rare diseases such as dystrophinopathies. Trial readiness not only involves conducting natural history studies and identifying clinically meaningful, sensitive outcome measures, but also encompassing feasibility assessment and the identification of participants’ eligibility for specific protocols. By use of the data collected in the yearly questionnaires (level 2), an initial screening for common inclusion and/or exclusion criteria, such as medication use and the reach of specific milestones, is possible. Validation of genetic results is essential due to the fact that many therapies for DMD target only a specific subset of the population. To ensure significant participant numbers for dystrophinopathy trials, a global clinical trial network is in place (TREAT-NMD), with DDD as a core member of the TREAT-NMD Global Registry Network. So, DDD significantly contributes by allowing to assess the feasibility of clinical trials, identify target (sub)populations, and facilitate the recruitment of eligible candidates.

The design and governance of DDD was carefully thought out to facilitate collaboration with different parties. Registration levels 4 and 5 were designed in adherence to GDPR guidelines [52], to facilitate data exchange with both academic and non-academic partners, including pharmaceutical companies. It’s noteworthy that a considerable proportion of the participants did not provide consent to share coded individual data with commercial partners. To date, DCN has not received any individual data sharing request from pharmaceutical companies. Thus, the actual usability of level 5 is still to be explored. Additionally, the rigid distinction between commercial and non-commercial proves an oversimplification of the current research landscape. It may require redefinition in future versions of the protocol and patient information forms. Considering that the decision to share data could depend on the nature of the request from a commercial partner, a dynamic consent model for (semi)commercial requests, as opposed to the current broad consent model, might be a more practical solution. This approach would also empower patients by providing added control over their participation in commercial studies.

The governance structure, including the DDD general- and advisory board, along with semi-annual newsletters to DDD participants, ensures transparency and effective utilization of the data. To enhance semantic interoperability, the functional design of the registry was governed by an information framework (FISMA). FISMA defines the context of data elements, demarcating and annotating each parameter with internationally agreed, ontology-based classifications [26]. The use of annotations, standardized protocols, and uniform language, aligned with FAIR principles, makes it easier to synchronize or exchange data with (inter)national dystrophinopathy RWD initiatives. To elaborate further, we aim to implement federated FAIR inquiries within DDD [26, 51, 53, 54]. Currently, the implementation of a semi-automated pipeline designed to address and integrate the results of pre-approved queries from independent registries is underway, with as ultimate goal, a dynamically accessible FAIR data element pool. This process occurs within a secure, privacy-sensitive environment.

DDD also has limitations. Prioritizing quality over quantity, the focus is on acquiring high-quality standardized data from patients at the DCN experts’ centers. Currently, clinical data from patients at other academic hospitals in the Netherlands is not collected (registration level 3). Additionally, adult patients with DMD sometimes only visit one of four CHMVs in the Netherlands [30]. As these CHMVs are part of four other Dutch academic hospitals, rather than LUMC and Radboudumc, clinical data from CHMVs requires considerable effort from data managers to be added to the registry potentially leading to missing data.

Adult BMD patients, especially at the milder end of the spectrum, often do not receive annual follow-up in an expert center, and thus, do not undergo a full functional assessment. To address this limitation, a national biobank for DMD and BMD is being established, collecting anonymized data from all academic hospitals and CHMVs.

To ensure interoperability, the information model & framework FISMA, which underlies DDD, also serves as a guideline for this national biobank.

Another limitation is that the current implementation of DDD does not include patient-reported outcome measure (PROM) scales like the PROM-UL or quality of life assessments. Given the increasing importance of PROMs in clinical practice and pharmaceutical trials, future iterations of DDD should consider including these.

In collaboration with Foundation 29, the Dutch Duchenne Parent Project has established a Duchenne Data Platform to empower patients and their caregivers [55]. This platform enables patients to access their health information securely, with data from various healthcare institutions stored in a manner that ensures exclusive access for the patient. Moreover, the Duchenne data platform will serve for patient preference studies, and efforts are underway to facilitate data exchange between DDD and this platform.

DDD has been designed to ensure standardized, longitudinal high-quality data within its clinical context, whilst minimizing the registration burden for patients and clinicians. It is important to recognize that establishing a multicenter interoperable database is a time-intensive process. In 2018 we started by engaging with stakeholders and designing the information model. However, it wasn’t until 2020 that the first patients could be included. A significant challenge remains in ensuring the sustainability and maintenance of this registry. This demands long-term financial backing and personnel support. Continuous evaluation of processes and filling of content, along with on-going efforts to improve and align DDD with international initiatives and emerging questions, are essential to ensure the registry’s long-term viability and relevance.

Actively including as many patients as possible with longitudinal follow-up demands a sustained commitment and hands-on coordination. An example highlighting the challenge is the re-registration process from the second to the third version of DDD, where 20% of patients did not respond despite multiple efforts, such as email, letters, and phone contact. Also maintaining up-to-date contact details and deceased status, is an ongoing challenge requiring careful attention.

Future innovations, like the curation of health information by artificial intelligence, may also be beneficial. However, we foresee that sustained funding remains essential for ensuring high-quality data collection and effective use of RWD, especially considering the limited availability of clinicians and the continuous need for inclusions of participants, adaptations, monitoring, and quality control.

## CONCLUSION

The third version of DDD utilizes a dual structure to capture both patient-reported and standardized healthcare data with minimal registration burden for patients and clinicians. DDD has proven useful to select and approach eligible participants for clinical trials and natural history studies, to gain insight into epidemiology and disease progression, and to facilitate conditional reimbursement of drugs in the Netherlands. Furthermore, by building on a common framework, we have successfully demonstrated the possibility to capture and exchange high-quality, interoperable, RWD. Our study also demonstrates the extra effort needed to sustain registries: this endeavor should be considered an essential part of both current clinical care, and the implementation of therapies. Therefore, future efforts need to focus on further improving the coverage, the interoperability, and ongoing assurance of data quality, and to capture the most valuable data, potentially includingPROMS.

## Data Availability

The data supporting the findings of this study are available on request from the corresponding author. The data are not publicly available due to privacy or ethical restrictions.
